# Combination of disease burden before allogeneic transplantation and early post-transplant minimal residual disease predicts survival in patients with acute myeloid leukemia

**DOI:** 10.1007/s00277-025-06325-x

**Published:** 2025-04-25

**Authors:** Claudia Núñez-Torrón Stock, Carlos Jiménez Chillón, Clara López Hernández, Fernando Martín Moro, Juan Marquet Palomanes, Miguel Piris Villaespesa, Alejandro Luna de Abia, Ernesto Roldán Santiago, Eulalia Rodríguez Martín, Anabelle Chinea Rodríguez, Valentín García Gutiérrez, Gemma Moreno Jiménez, Javier López Jiménez, Pilar Herrera Puente

**Affiliations:** 1 Departamento de Hematología y Hemoterapia, Hospital Universitario Infanta Sofía, Avenida Paseo de Europa, Madrid, 34 28702 Spain; 2 Universidad Alcalá de Henares, Madrid, Spain; 3 Universidad Europea de Madrid, Madrid, Spain; 4 Hematology and Hemotherapy Service, Instituto Ramón y Cajal de Investigación Sanitaria (IRYCIS), Hospital Universitario Ramón y Cajal, Madrid, Spain; 5 Departamento de Hematología y Hemoterapia, Hospital Universitario Gregorio Marañón, Madrid, Spain; 6 Departamento de Hematología y Hemoterapia, Hospital Universitario Ramón y Cajal, Madrid, Spain; 7 Departamento de Inmunología, Hospital Universitario Ramón y Cajal, Madrid, Spain

**Keywords:** Acute myeloid leukemia, Allogeneic transplantation, Pretrasplantation disease burden, Minimal residual disease, multiparameter flow cytometry

## Abstract

The burden disease before allogeneic transplantation (HSCT) or the early post-transplant minimal residual disease (MRD) are both predictive parameters for relapse and post-HSCT survival in acute myeloid leukemia (AML). Nonetheless, the combination of both can provide more accurate information to identify high risk patients. To analyze the impact of pre-HSCT disease burden (MRD- vs. MRD + vs. active disease (AD), the early post-transplant MRD (posMRD + vs. posMRD-), and the combination of both pre- and post-HSCT disease status of the post-HSCT outcomes in AML patients. We retrospectively analyzed 173 patients with AML who underwent HSCT in a single institution, patients were classified according to pre-HSCT disease status, and post-HSCT MRD. MRD was measured by multiparameter flow cytometry using a cut-off of 0.1% for MRD+. The post-HSCT outcomes were analyzed based on the pre-transplant status, post-transplant status, and by combining both parameters. Patients with AD and MRD + before HSCT had worse 3y-event free (EFS) and overall survival (OS) than MRD- patients, due to a higher cumulative incidence of relapse (CIR). Also, patients with posMRD + had worse outcomes than posMRD- group. In the combined analysis, patient with MRD-/posMRD- had the best EFS and OS (3y-EFS 66.5%, 3y-OS 70.0%). Patients with MRD+/posMRD- have worse prognosis (3y-EFS 39.0%, 3y-OS 54.0%) and specially the group with AD/MRD- (3y-EFS 13.5%, 3y-OS 22.0%) and posMRD + regardless pre-HSCT disease status(3y-EFS 26.5%, 3y-OS 28.0%) had dismal OS and EFS. The combination of pre-HSCT disease burden and post-HSCT MRD measurements help us for identifying high-risk subgroups. Any level of pre-transplant disease (MRD+, and especially patients with active AD) is a risk factor, even when MRD- was achieved post-transplant. Patients with post-transplant MRD + also had an adverse prognosis. These should be target groups for implementing tailored pre- and post-transplant strategies to improve outcomes.

**Supplementary Information:**

The online version contains supplementary material available at 10.1007/s00277-025-06325-x.

## Introduction

In patients with acute myeloid leukemia (AML), allogeneic hematopoietic stem cell transplantation (HSCT) represents the primary curative strategy for most patients with intermediate or adverse risk, as well as for those with low risk who do not achieve complete remission (CR) with negative minimal residual disease (MRD) following intensive chemotherapy treatment. Despite this, post-transplant relapse remains the leading cause of treatment failure [1].

The disease burden prior to HSCT is one of the most critical factors influencing post-transplant survival, as we demonstrated in our cohort’s previous studies [2, 3]. Patients undergoing HSCT in refractory/relapse disease state have a poorer prognosis compared to those who achieve CR before HSCT [1, 2]. Among patients in CR, pre-transplant positive MRD has been identified as an independent adverse factor for survival [3, 4]. Several techniques are available for MRD measurement, being multiparameter flow cytometry (MFC) one of the most widely used due to its wider applicability.

Regarding the disease burden prior to transplantation, it appears that a higher disease burden prior to transplantation worsens survival [5, 6]. However, some studies, such as that by Araki et al., have observed similar post-HSCT survival rates when comparing patients with positive MRD to those with AD [7].

Although few studies report the influence of MRD in early post-transplant outcomes, MRD assessment during this period can help to identify high-risk patients with an unfavourable prognosis [8–20].

Furthermore, the combination of pre- and post-transplant MRD measurements may provide a more accurate risk stratification for relapse than evaluating either parameter individually [21–24]. This study aimed to analyse the impact of pre-HSCT disease burden and early post-HSCT MRD by MFC on post-HSCT survival and relapse incidence, both individually and by combining the two parameters.

## Materials and methods

The study protocol and informed consent process was reviewed and approved by the Ramón y Cajal Hospital Ethics Committee (243/21), and the study was conducted in accordance with the Declaration of Helsinki.

### Study population and variables

We performed a retrospective analysis of 173 consecutive adult patients (≥ 18 years) diagnosed with AML, who received HSCT between 1st January 2008 and 31st Dec 2023 at Ramón y Cajal Hospital in Madrid. Disease-related variables included AML subtype according to the WHO 2016 classification [25], genetic risk according to the European LeukemiaNet 2017 risk classification [26], presence of complex karyotype (CK) and monosomal karyotype (MK) at diagnosis. Transplant-related variables included in the analysis were disease burden (AD, CR and MRD status by MFC before HSCT), HCT-CI score, time from diagnosis to HSCT, conditioning intensity, conditioning scheme, graft-vs-host disease prophylaxis scheme, donor/recipient CMV serologic status, donor source, donor HLA type, HLA matching, median infused CD34+/kg and MRD status after HSCT.

### Bone marrow samples and MRD assessment by MFC

All patients had an assessable bone marrow before HSCT (including patients in CR with evaluable MRD by MFC or patients transplanted with AD) and/or at least one available MRD measurement within days + 30 and + 100 after HSCT. MRD assessment by MFC was performed as previously described [2]. Positive MRD was defined as ≥ 0.1% following ELN recommendations, as reported elsewhere [2, 27].

### Conditioning scheme and GHVD prophylaxis

Conditioning intensity was chosen according to the institutional strategy, considering age and comorbidities of the patient at the time of transplantation. Although MRD status before transplant was known to clinicians, it was not an influential factor in the choice of conditioning intensity and only patients transplanted with AD were proposed to intensification with sequential conditioning when possible. The conditioning scheme was cyclophosphamide and busulfan-based between 2008 and 2011, and, since 2011, fludarabine and busulfan-based. As previously reported, RIC was defined as a total busulfan dose of less than 9 mg/kg iv [28]. Graft-versus-host disease (GVHD) prophylaxis included cyclosporine (CsA) and mycophenolate (MMF) for RIC, CsA and methotrexate for MAC and post-transplant cyclophosphamide (PTCy), CsA and MMF for haploidentical transplant. Since 2019, PTCy was added as GvHD prophylaxis for patients with one or more HLA mismatches. Since 2023, irrespective of HLA compatibility, all patients received PTCy, tacrolimus and MMF as GvHD prophylaxis. Thymoglobulin and thiotepa were used in all patients with an unrelated donor but since 2023 the use of ATG was discontinued. Thymoglobulin dose was 6 mg/kg iv for both MAC and RIC. For patients who received MAC, thiotepa dose was 10 mg/kg iv and in those cases busulfan total dose was adjusted to 9.6 mg/kg iv. For patients who received RIC, thiotepa dose was 5 mg/kg iv and busulfan 6.4 mg/kg iv. Peripheral blood (PB) was the source of progenitor cells for all patients. The post-HSCT maintenance pharmacological strategy (excluding treatment in the context of overt hematologic relapse) has been established for high-risk patients at the center since 2021. This strategy involves the use of hypomethylating agents and/or FLT3-ITD inhibitors +/- donor lymphocyte infusion depending on patient´s mutations and clinical condition. Prior to this period, only two isolated cases with FLT3-ITD positive who received off-label maintenance treatment with sorafenib.

### Clinical endpoints and definitions

The primary endpoints of the study were cumulative incidence of relapse (CIR) and event-free survival (EFS). Other endpoints of interest were overall survival (OS) and transplant-related mortality (TRM). We defined CR as < 5% blasts on bone marrow cytology with no circulating blasts in PB and absence of extramedullary disease. OS was defined as the time from transplantation to death and EFS as the time from transplantation to either relapse or death. Relapse was considered as reappearance of ≥ 5% blasts in bone marrow, circulating blasts or extramedullary disease. CIR was defined as time to onset of leukemia recurrence. TRM was defined as death without relapse [26, 29].

### Statistical analysis

Chi-squared test or Fisher´s test were used to compare differences between categorical variables and Student’s T or Mann-Whitney U test for continuous variables. OS and EFS were estimated using the Kaplan-Meier method and differences were analyzed using the log rank test. The Holm-Bonferroni correction was applied to multiple comparisons. CIR and non-relapse mortality were estimated using cumulative incidence method and differences were estimated using Gray´s method, considering each risk as a competing risk. All *p*-values were two-sided and *p* < 0.05 was considered significant. Multivariate analyses were performed using Cox proportional hazards model for OS and EFS, and Fine–Gray proportional hazard regression was used for CIR including the variable combined pre- and post-HSCT disease status (MRD-/posMRD-; MRD+/posMRD-; AD/posMRD-; posMRD+) and adjusting for clinical and sociodemographic characteristics. SPSS v.22 (IBM) and XLSTAT 2020.5.1 softwares were used to perform the statistical analysis.

## Results

The median age of overall cohort (*n* = 173 patients) was 54 [interquartile range 45–61] years, and 58.4% were males. At AML diagnosis, 18.0% of patients had favourable, 54.1% intermediate and 27.9% adverse risk by ELN2017 classification. Fifteen patients (8.7%) had MK and 23 (14.4%) CK. Most patients (68.2%) were transplanted in first CR (CR1). The donor was sibling donor in 36.4%, haploidentical in 37% and unrelated in 26.6%. The conditioning intensity was myeloablative in 52.6%, 42.8% reduce intensity and 4.6% sequential conditioning.

**Fig. 1:**
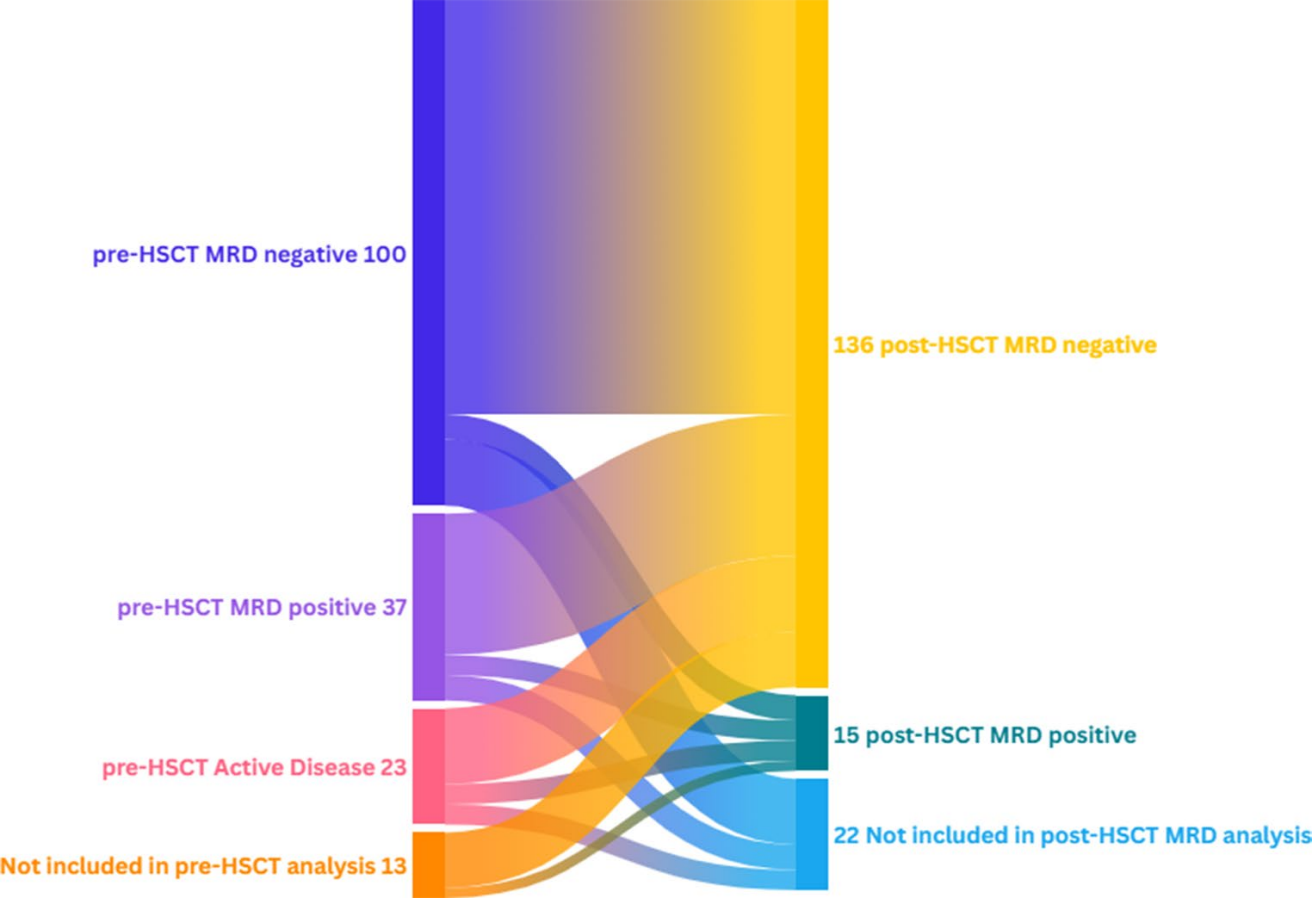
Sankey diagram of patients with LMA included in the study. On the left of the diagram patients are classified according to the pre-HSCT disease status, while on the right patients are classified according to early post-HSCT MRD. HSCT, haematopoietic stem cell transplantation; MRD, minimal residual disease

According to pre-HSCT disease, 160 patients had an assessable bone marrow: 100 (62.5%) patients in CR with MRD-, 37 (23.1%) in CR with MRD + and 23 (14.3%) with AD. Thirteen patients were not included in the pre-HSCT analysis because they were transplanted in aplasia (*n* = 7), or they had not an assessable bone marrow (*n* = 6). According to early post-HSCT, 151 patients had at least one assessable bone marrow aspiration in CR with valuable MRD (posMRD), of those 136 had posMRD- (90.1%) and 15 posMRD+ (9.9%). Patients not included in the early post-HSCT MRD evaluation were due to AD at first evaluation (*n* = 3), hypoplastic bone marrow with not assessable MRD (*n* = 7), premature death without evaluation (*n* = 9) and 3 patients in whom, due to medical complications in the post-transplant period, the first bone marrow was performed outside the period established in this study. Finally, we combined pre- and post-HSCT disease status (*n* = 140) and divided patients among four different groups according to MRD dynamics. Due to the small number of post-HSCT MRD + patients, we grouped all of them into a single group for the analysis of the impact of combining pre- and post-transplant disease. However, a sub analysis was conducted for patients with post-HSCT MRD + and available pre-HSCT disease status. The four established groups were as follows: (1) patients with MRD-/posMRD- (*n* = 82) (58.6%), (2) patients with MRD+/posMRD- (*n* = 28) (20.0%), (3) patients with AD/posMRD- (*n* = 15) (12.1%) and finally (4) patients with posMRD + regardless of the pre-HSCT status (posMRD+) (*n* = 15) (12.1%). In the latter, pre-transplant disease status was available for 13 of the patients. Four patients had AD before HSCT, 4 were MRD + pre- and post-HSCT and 5 progressed from MRD- to posMRD+ **(**Fig. 1**).** The baseline characteristics of overall cohort, pre-HSCT, post-HSCT and pre/post HSCT disease are reflected in Supplementary Table 1. The median follow-up was 13 [6.0-15.5] months in overall population and 48 [15.0-90.8] months in survivors.

### Event-free survival and overall survival

**Fig. 2:**
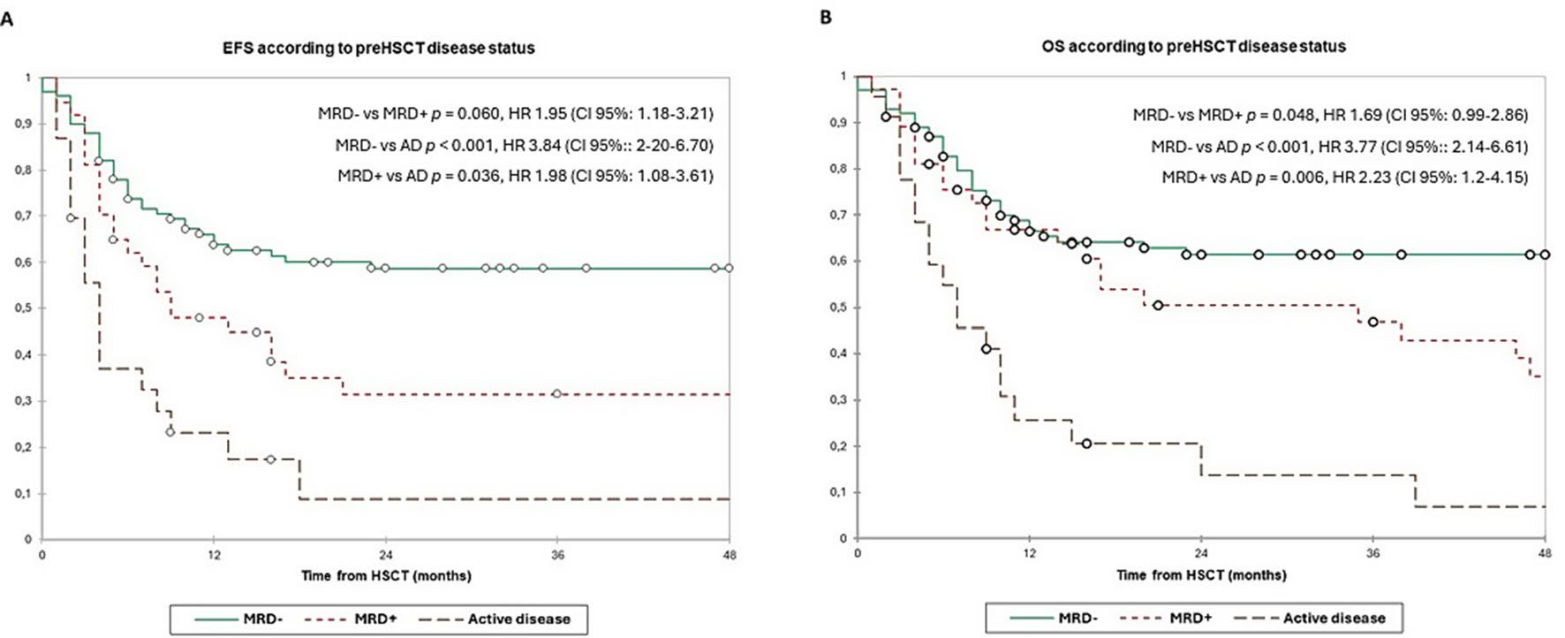
Event-free survival (1 A) and overall survival (1B) according to pre-HSCT disease burden. Estimates of (left) EFS and (right) OS after HSCT for patients with AML according to pre-HSCT, shown individually for MRD- (*n* = 100), MRD+ (*n* = 37) and AD (*n* = 23) respectively. Patients with MRD + and especially those with AD have significantly worse EFS and OS than patients with MRD- (3y-EFS 58.5% in MRD- vs. 31.5% in MRD + vs. 9.0% in AD patients; 3y-OS 61.5% vs. 47.0% vs. 13.5%). AD, active disease; EFS, event-free survival; HSCT, haematopoietic stem cell transplantation; MRD, minimal residual disease; OS, overall survival

**Fig. 3:**
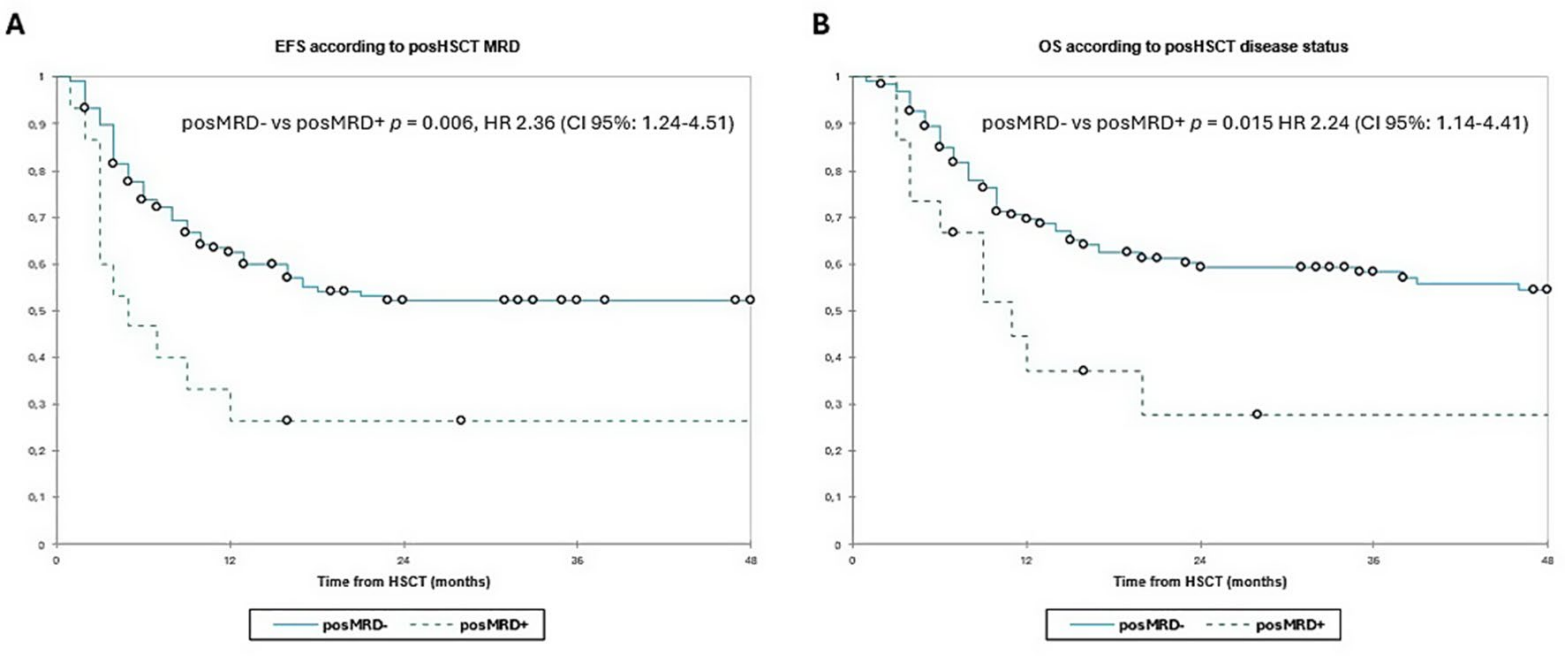
Event-free survival (2 A) and overall survival (2B) according to post-HSCT disease burden. Estimates of (left) EFS and (right) OS after HSCT for patients with AML according to post-HSCT, shown individually for posMRD- (*n* = 136) and posMRD+ (*n* = 15) respectively. Patients with posMRD + have significantly worse EFS and OS than patients with posMRD- (3y-EFS 52.0% in posMRD- vs. 26.5%% in MRD+; 3y-OS 58.0% vs. 28.0%). EFS, event-free survival; HSCT, haematopoietic stem cell transplantation; MRD, minimal residual disease; OS, overall survival

**Fig. 4:**
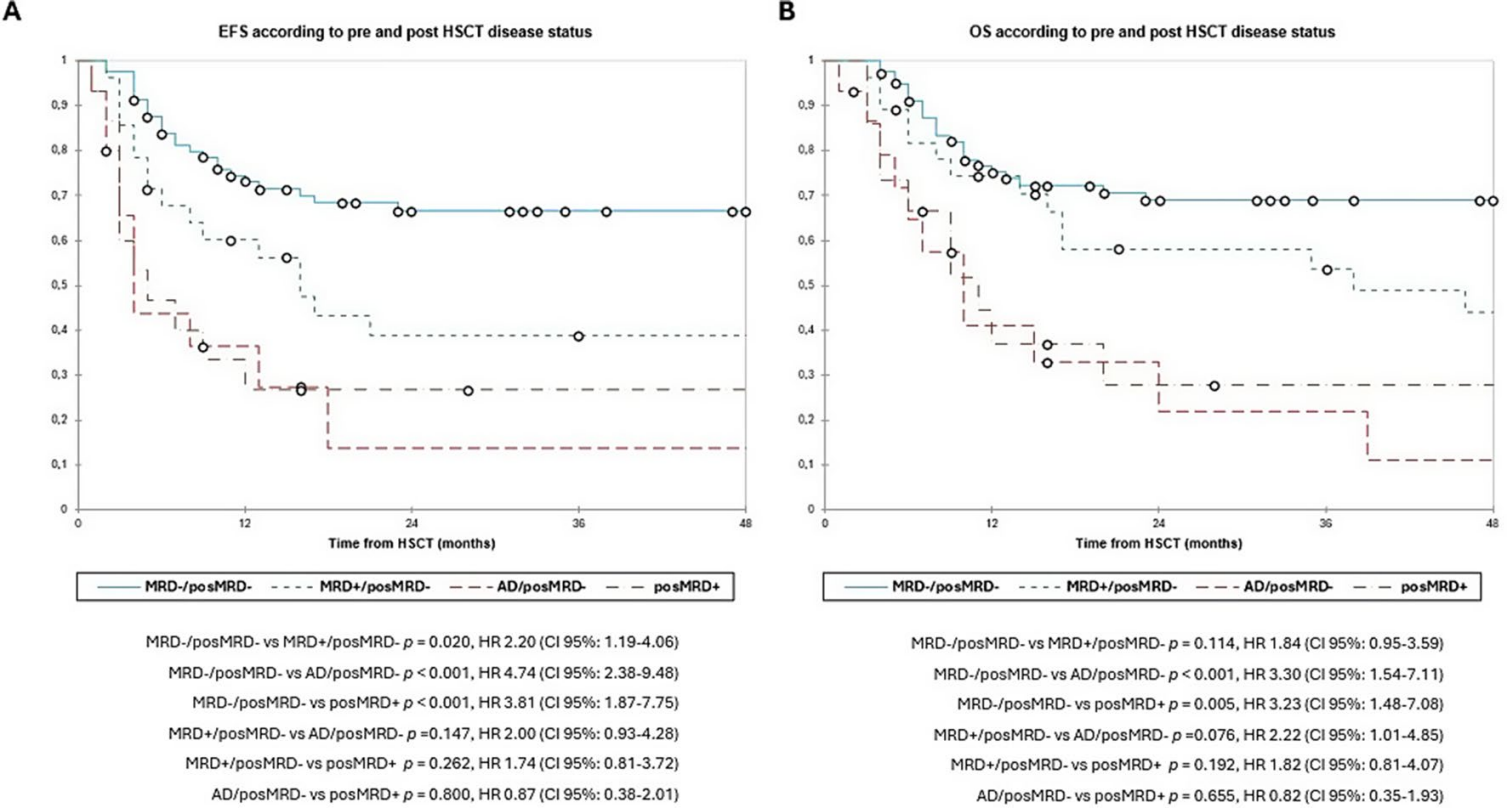
Event-free survival (2 A) and overall survival (2B) according to combined pre- and post-HSCT disease burden. Estimates of (left) EFS and (right) OS after HSCT for patients with AML according to combined pre-HSCT and post-HSCT, shown individually for MRD-/posMRD- (*n* = 82), MRD+/posMRD- (*n* = 28), AD/posMRD- (*n* = 15) and posMRD+ (*n* = 15) respectively. While patients with MRD-/posMRD- have the best prognosis, patients with MRD+/posMRD- have an intermediate prognosis and patients with AD/MRD- and posMRD + have the worst EFS and OS (3y-EFS was 66.5% in MRD-/posMRD- vs. 39.0% in MRD+/posMRD- vs. 13.5% in AD/MRD- vs. 26.5% posMRD+; 3y-OS was 70.0% vs. 54.0% vs. 22.0% vs. 28.0%). AD, active disease; EFS, event-free survival; HSCT, haematopoietic stem cell transplantation; MRD, minimal residual disease; OS, overall survival

The 3y-EFS was 44.5% and 3y-OS was 50.5% in overall population. According to pre-HSCT disease, the 3y-EFS was 58.5% in MRD-, 31.5% in MRD + and 9.0% in AD patients (MRD- vs. MRD + *p* = 0.060; MRD- vs. AD + *p* < 0.001; MRD + vs. AD *p* = 0.036). The 3y-OS was 61.5%, 47.0% and 13.5% respectively (MRD- vs. MRD + *p* = 0.048; MRD- vs. AD *p* < 0.001; MRD + vs. AD *p* = 0.006, **(**Fig. 2A and B**)**. Then, we evaluated the survival according to early post-HSCT MRD status, reporting a 3y-EFS of 52.0% vs. 26.5% (*p* = 0.006, and 3y-OS 58.0% vs. 28.0% (*p* = 0.015, (Fig. 3A and B). Finally, we evaluated the combined pre- and post-HSCT disease status. The 3y-EFS was higher in patients with MRD-/posMRD- (66.5%), compared to MRD+/posMRD- patients (39.0%) and specially the AD/MRD- and posMRD + groups, who had a 3y-EFS of 13.5% and 26.5% respectively (MRD-/posMRD- vs. MRD+/posMRD- *p* = 0.020; MRD-/posMRD- vs. AD/posMRD- *p* < 0.001; MRD-/posMRD- vs. posMRD + *p <* 0.001; MRD+/posMRD- vs. AD/posMRD- *p* = 0.147; MRD+/posMRD- vs. posMRD + *p* = 0.262; AD/posMRD- vs. posMRD + *p* = 0.800. Also the 3y-OS was better in MRD-/posMRD- (70.0%) compared to MRD+/posMRD- (54.0%) and both groups had better OS than AD/posMRD- patients (22.0%) and posMRD + group (28.0%) (MRD-/posMRD- vs. MRD+/posMRD- *p* = 0.114; MRD-/posMRD- vs. AD/posMRD- *p <* 0.001; MRD-/posMRD- vs. posMRD + *p =* 0.005; MRD+/posMRD- vs. AD/posMRD- *p* = 0.076; MRD+/posMRD- vs. posMRD + *p* = 0.192 and AD/posMRD- vs. posMRD + *p* = 0.655) **(**Fig. 4A and B**)**.

We analysed the 13 patients with known pre-HSCT disease who were posMRD+. Patients with posMRD + and detectable pre-transplant disease (both active disease or MRD+) had worse outcomes than patients with posMRD + who proceeded to allograft with a negative MRD (EFS: AD/MRD + vs. MRD+/posMRD + *p* = 0.282; AD/MRD + vs. MRD-/posMRD + *p* = 0.046; MRD+/posMRD + vs. MRD-/posMRD + *p* = 0.368) (OS: AD/MRD + vs. MRD+/posMRD + *p* = 0.120; AD/MRD + vs. MRD-/posMRD + *p* = 0.074; MRD+/posMRD + vs. MRD-/posMRD + *p* = 0.916) **(Supplementary Figure SF1A and SF1B).**

We conducted both univariate analysis and a multivariate analysis for both EFS and OS for the 140 patients with available pre- and post-HSCT disease **(**Tables 1 and 2**)**. In the multivariate analysis, variables with a significant HR for EFS were a MPN Ph- in blastic phase [HR 50.80 (9.44–273.31)], MK [HR 2.36 (1.07–5.20)], the disease status MRD+/posMRD- [HR 2.18 (1.16–4.11)], AD/posMRD- [HR 4.59 (2.16–9.75)] and posMRD+ [HR 3.38 (1.54–7.46)]. Variables with a significant HR for OS were AML with myelodysplasia related changes [HR 2.32 (1.14–4.71)], disease status AD/MRD- [HR 3.30 (1.54–7.11)] or posMRD+ [HR 3.23 (1.48–7.08)].

**Table 1:** Univariate and multivariate analysis for event-free survival for combined pre- and post-HSCT disease *n* = 140

Variable	Univariate analysis		Multivariate analysis	
	Hazard ratio (CI 95%)	*p* value	Hazard ratio (CI 95%)	*p* value
**Male**				
[ref] No				
Yes	0.81 (0.50–1.31)	0.387		
**WHO 2016 classification**				
[ref] Recurrent genetic abnormalities				
Myelodysplasia-related changes	**1.99 (1.05–3.77)**	0.034*	1.43 (0.72–2.85)	0.306
Therapy-related	1.92 (0.83–4.45)	0.127	1.17 (0.48–2.90)	0.728
Not otherwise specified	0.81 (0.37–1.78)	0.601	0.97 (0.44–2.15)	0.934
Blastic phase of MPN Ph-	**27.44 (5.31–141.97)**	< 0.001*	**50.80 (9.44–273.31)**	< 0.001*
**ELN 2017 risk classification#**				
[ref] Favourable risk				
Intermediate risk	1.27 (0.60–2.70)	0.529		
Adverse risk	**2.47 (1.16–5.29)**	0.020*		
**Complex karyotype#**				
[ref] No				
Yes	**2.57 (1.42–4.67)**	0.002*		
**Monosomal karyotype**				
[ref] No				
Yes	**3.22 (1.62–6.41)**	< 0.001*	**2.36 (1.07–5.20)**	0.033*
**CR status before HSCT**				
[ref] CR1				
>= CR2	0.80 (0.34–1.88)	0.606		
**HCT-CI score at HSCT**				
[ref] < 3				
>= 3	1.53 (0.87–2.73)	0.149		
**Conditioning intensity**				
[ref] Myeloablative				
Reduced intensity	1.21 (0.72–2.02)	0.473		
**Donor type**				
[ref] Related donor				
Unrelated donor	0.62 (0.31–1.24)	0.179		
Haploidentical donor	0.99 (0.58–1.68)	0.971		
**HLA mismatch**				
[ref] No				
Yes	1.17 (0.52–2.63)	0.706		
**CMV status (D/R)**				
[ref] Neg/neg				
Other	0.52 (0.22–1.21)	0.129		
**Age at HSCT**				
[ref] < 60 years				
>= 60 years	1.00 (0.59–1.670)	0.998		
**Pre and postHSCT disease**				
[ref] MRD-/posMRD-				
MRD+/posMRD-	**2.20 (1.19–4.06)**	0.012*	**2.18 (1.16–4.11)**	0.016*
AD/MRD-	**4.74 (2.38–9.48)**	< 0.001*	**4.59 (2.16–9.75)**	< 0.001*
posMRD+	**3.81 (1.87–7.75)**	< 0.001*	**3.38 (1.54–7.46)**	0.003*

* *p* value < 0.05; AD, active disease; CMV, cytomegalovirus; CR1, first complete remission; CR2, second complete remission; ELN, European LeukemiaNet; HCT-CI haematopoietic cell transplantation-specific comorbidity index. HSCT, haematopoietic stem cell transplantation; MRD, minimal residual disease; MFC, multiparameter flow cytometry; MPN Ph-, myeloproliferative neoplasm Philadelphia negative; NOS, not otherwise specified; WHO, World Health Organisation

&variables with a *p* value < 0.100 and those that are confounding were included

#Variables were not included in the multivariate analysis due to high collinearity with the monosomic karyotype variable

**Table 2:** Univariate and multivariate analysis for overall survival for combined pre- and post-HSCT disease *n* = 140

Variable	Univariate analysis		Multivariate analysis	
	Hazard ratio (CI 95%)	*p* value	Hazard ratio (CI 95%)	*p* value
Male				
[ref] No				
Yes	0.68 (0.41–1.14)	0.145		
**WHO 2016 classification**				
[ref] Recurrent genetic abnormalities				
Myelodysplasia-related changes	**2.56 (1.27–5.17)**	0.009*	**2.32 (1.14–4.71)**	0.020*
Therapy-related	**2.83 (1.17–6.83)**	0.021*	2.38 (0.96–5.90)	0.063
Not otherwise specified	1.06 (0.45–2.50)	0.895	1.31 (0.55–3.14)	0.543
Blastic phase of MPN Ph-	3.38 (0.43–26.41)	0.246	5.34 (0.66–42.93)	0.115
**ELN 2017 risk classification#**				
[ref] Favourable risk				
Intermediate risk	1.69 (0.74–3.87)	0.215	1.20 (0.40–3.67)	0.744
Adverse risk	**2.82 (1.21–6.57)**	0.016*	1.62 (0.52–5.01)	0.407
**Complex karyotype#**				
[ref] No				
Yes	**2.26 (1.22–4.18)**	0.010*	0.59 (0.16–2.23)	0.437
**Monosomal karyotype**				
[ref] No				
Yes	**3.18 (1.60–6.31)**	< 0.001*	1.94 (0.90–4.16)	0.091
**CR status before HSCT**				
[ref] CR1				
>= CR2	0.97 (0.41–2.30)	0.947		
**HCT-CI score at HSCT**				
[ref] < 3				
>= 3	1.55 (0.84–2.89)	0.164		
**Conditioning intensity**				
[ref] Myeloablative				
Reduced intensity	1.48 (0.85–2.55)	0.164		
**Donor type**				
[ref] Related donor				
Unrelated donor	0.68 (0.34–1.37)	0.283		
Haploidentical donor	0.71 (0.40–1.27)	0.247		
**HLA mismatch**				
[ref] No				
Yes	0.97 (0.41–2.32)	0.954		
**CMV status (D/R)**				
[ref] Neg/neg				
Other	0.82 (0.29–2.26)	0.696		
**Age at HSCT**				
[ref] < 60 years				
>= 60 years	1.28 (0.74–2.22)	0.377		
**Pre and postHSCT disease**				
[ref] MRD-/posMRD-				
MRD+/posMRD-	**1.99 (1.04–3.81)**	0.038*	1.84 (0.95–3.59)	0.073
AD/MRD-	**3.76 (1.83–7.73)**	< 0.001*	**3.30 (1.54–7.11)**	0.002*
posMRD+	**3.59 (1.70–7.57)**	< 0.001*	**3.23 (1.48–7.08)**	0.003*

* *p* value < 0.05; AD, active disease; CMV, cytomegalovirus; CR1, first complete remission; CR2, second complete remission; ELN, European LeukemiaNet; HCT-CI haematopoietic cell transplantation-specific comorbidity index. HSCT, haematopoietic stem cell transplantation; MRD, minimal residual disease; MFC, multiparameter flow cytometry; MPN Ph-, myeloproliferative neoplasm Philadelphia negative; NOS, not otherwise specified; WHO, World Health Organisation

&variables with a *p* value < 0.100 and those that are confounding were included

#Variables were not included in the multivariate analysis due to high collinearity with the monosomic karyotype variable

### Cumulative incidence of relapse and transplant-related mortality

In the overall cohort, 54 patients relapsed after HSCT. The median time from HSCT to relapse was 4 [3-8.25] months. Forty-six patients died after relapse during study follow-up, with a median time since relapse to death of 3 [1.25-4] months. Thirty-nine patients died without evidence of relapse. The main cause of death was infectious complications in 21 patients, GVHD in five, mixed (infectious complication and GVHD) in seven, cerebral oedema in one, veno-occlusive disease in one, solid tumour in one and fatal haemorrhage in another. The cause of death was unknown in two patients. The median time from HSCT to TRM was 4 [2–9] months.

**Fig. 5:**
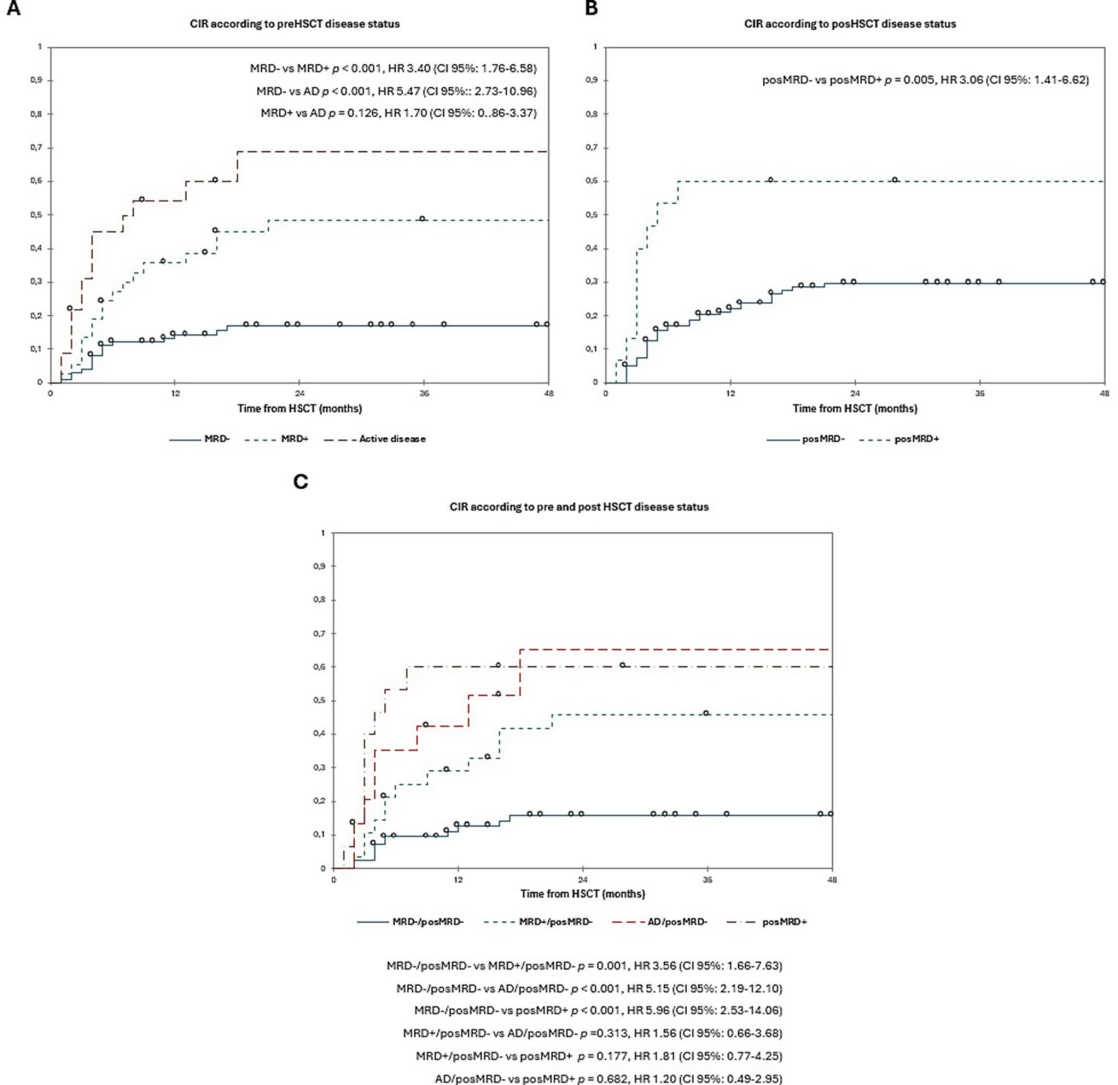
Cumulative incidence of relapse according to pre-HSCT status (4 A), post-HSCT MRD (4B) and combined pre- and post-HSCT disease burden (4 C). Estimates of CIR after HSCT for patients with AML according to pre-HSCT (up-left), shown individually for MRD- (*n* = 100), MRD+ (*n* = 37) and AD (*n* = 23) respectively. Estimates of CIR after HSCT for patients with AML according to post-HSCT (up-right) and combined pre and posHSCT disease burden (down-left), shown individually for posMRD- (*n* = 136) and posMRD+ (*n* = 15) respectively. Estimates of CIR after HSCT for patients with AML according to pre- and post-HSCT disease burden (down-left), according to combined pre-HSCT and post-HSCT, shown individually for MRD-/posMRD- (*n* = 82), MRD+/posMRD- (*n* = 28), AD/posMRD- (*n* = 15) and posMRD+ (*n* = 15) respectively. Patients with MRD + and especially those with AD have significantly worse CIR than patients with MRD- (3y-CIR 17.0% in MRD- vs. 49.0% in MRD + vs. 69.0% in AD patients). Also, patients with posMRD + have worse CIR than posMRD- (3y-CIR 30% in posMRD- vs. 60.0% in posMRD+). In combined group, patients with posMRD + and AD/MRD- have the higher CIR, while patients with MRD+/posMRD- have an intermediate prognosis and the MRD-/posMRD- have the best prognosis (3y-CIR 16.0% in MRD-/MRD- vs. 46.0% in MRD+/MRD- vs. 65.0% in AD/MRD- and 60.0% in posMRD + respectively). AD, active disease; CIR, cumulative incidence of relapse; HSCT, haematopoietic stem cell transplantation; MRD, minimal residual disease

The 3y-CIR was 33.0% in overall cohort. The 3y-CIR was 17.0% in MRD- 49.0% in MRD + and 69.0% in AD comparing the pre-HSCT status (*p* < 0.001) (MRD- vs. MRD + *p* < 0.001; MRD- vs. AD + *p* < 0.001; MRD + vs. AD *p* = 0.126). **(**Fig. 5A**)**. The 3y-CIR was 30.0% in posMRD- and 60.0% in posMRD+ (*p* = 0.005) **(**Fig. 5B**)**. In the combined pre- and post-HSCT disease status group, patients with MRD-/MRD- had the lowest 3y-CIR (16.0%), compared to 46.0% in patients with MRD+/MRD-. Those transplanted with AD/MRD-or posMRD + had the highest relapse rate, with a 3y-CIR of 65.0% and 60.0% respectively (MRD-/posMRD- vs. MRD+/posMRD- *p* = 0.001; MRD-/posMRD- vs. AD/posMRD- *p <* 0.001; MRD-/posMRD- vs. posMRD + *p* < 0.001; MRD+/posMRD- vs. AD/posMRD- *p* = 0.313; MRD+/posMRD- vs. posMRD + *p* = 0.177 and AD/posMRD- vs. posMRD + *p* = 0.682). **(**Fig. 5C**)**. In the multivariate analysis, variables with a significant HR for CIR were MPN Ph- in blastic phase [HR 87.55 (28.40–269.85)], the disease status MRD+/posMRD- [HR 3.62 (1.62–8.10)], AD/posMRD- [HR 5.61 (2.27–13.81)] and posMRD+ [HR 6.62 (2.35–18.70)] **(**Table 3**).**

**Table 3:** Univariate and multivariate analysis for cumulative incidence of relapse for combined pre- and post-HSCT disease *n* = 140

Variable	Univariate analysis		Multivariate analysis&	
	Hazard ratio (CI 95%)	*p* value	Hazard ratio (CI 95%)	*p* value
**Male**				
[ref] No				
Yes	0.74 (0.41–1.34)	0.314		
**WHO 2016 classification**				
[ref] Recurrent genetic abnormalities				
Myelodysplasia-related changes	1.86 (0.86–4.05)	0.115	1.31 (0.52–3.26)	0.567
Therapy-related	1.77 (0.65–4.85)	0.265	0.92 (0.30–2.77)	0.877
Not otherwise specified	0.54 (0.19–1.60)	0.263	0.60 (0.19–1.93)	0.393
Blastic phase of MPN Ph-	**34.87 (12.36–98.35)**	< 0.001*	**87.54 (28.40–269.85)**	< 0.001*
**ELN 2017 risk classification#**				
[ref] Favourable risk				
Intermediate risk	1.40 (0.46–4.28)	0.552		
Adverse risk	**4.48 (1.50–13.34)**	0.007*		
**Complex karyotype#**				
[ref] No				
Yes	**3.61 (1.91–6.84)**	< 0.001*		
**Monosomal karyotype**				
[ref] No				
Yes	**3.56 (1.72–7.37)**	0.001*	2.23 (0.85–5.86)	0.102
**CR status before HSCT**				
[ref] CR1				
>= CR2	0.64 (0.20–2.04)	0.448		
**HCT-CI score at HSCT**				
[ref] < 3				
>= 3	1.26 (0.62–2.59)	0.522		
**Conditioning intensity**				
[ref] Myeloablative				
Reduced intensity	1.30 (0.69–2.45)	0.417		
**Donor type**				
[ref] Related donor				
Unrelated donor	0.69 (0.30–1.62)	0.396		
Haploidentical donor	1.15 (0.59–2.21)	0.682		
**HLA mismatch**				
[ref] No				
Yes	1.50 (0.58–3.85)	0.402		
**CMV status (D/R)**				
[ref] Neg/neg				
Other	0.51 (0.19–1.35)	0.175		
**Age at HSCT**				
[ref] < 60 years				
>= 60 years	0.68 (0.34–1.36)	0.276		
**Pre and postHSCT disease**				
[ref] MRD-/posMRD-				
MRD+/posMRD-	**3.51 (1.66–7.42)**	0.001*	**3.62 (1.62–8.10)**	0.002*
AD/MRD-	**5.24 (2.20–12.46)**	< 0.001*	**5.60 (2.27–13.82)**	< 0.001*
posMRD+	**6.33 (2.54–15.75)**	< 0.001*	**6.62 (2.35–18.70)**	< 0.001*

* *p* value < 0.05; AD, active disease; CMV, cytomegalovirus; CR1, first complete remission; CR2, second complete remission; ELN, European LeukemiaNet; HCT-CI haematopoietic cell transplantation-specific comorbidity index. HSCT, haematopoietic stem cell transplantation; MRD, minimal residual disease; MFC, multiparameter flow cytometry; MPN Ph-, myeloproliferative neoplasm Philadelphia negative; NOS, not otherwise specified; WHO, World Health Organisation

&variables with a *p* value < 0.100 and those that are confounding were included

#Variables were not included in the multivariate analysis due to high collinearity with the monosomic karyotype variable

The 3y-TRM was 23.0% in overall cohort. The 3y-TRM was 24.5% in MRD- vs. 20.0% in MRD + vs. 22.5% in AD comparing the pre-HSCT status (*p* = 0.786) **(SF2A)**. The 3y-TRM was 18.0% in posMRD- and 13.0% in posMRD+ (*p* = 0.619) **(SF2B)**. In the group of patients with the combination of pre- and post-HSCT disease, the 3y-TRM was 17.5% in MRD-/MRD- vs. 15.0% in MRD+/MRD- vs. 21.0% in AD/posMRD- and 13.0% in posMRD + respectively (*p* = 0.847) **(SF2C)**.

## Discussion

In the present study, we present data from a cohort who was treated uniformly at the same center, providing a comprehensive view of the impact of pre-transplant disease burden, early post-transplant MRD by MFC on survival outcomes, as well as the combined effect of both measurements. Previous studies, including our published works focusing on pre-transplant disease, have demonstrated that both a positive MRD and the presence of AD prior to transplant have a negative impact on survival [2–4, 7, 30–45]. Although fewer studies exist in the field of early post-transplant, evidence—consistent with our series—suggests that positive residual disease in this context is highly indicative of a likely hematologic relapse [8–20].

Limited studies have examined the combined dynamics of pre- and post-transplant factors. Appelbaum et al. [22] studied transplanted patients in CR1/CR2 and separately analysed the influence on survival of pre-transplant MRD by MFC in one cohort and post-transplant MRD by MFC and FISH during the first through day 100 post-HSCT in a different cohort with overlapping characteristics. In the pre-HSCT MRD- cohort, they found 3y-OS of 73.0% (CR1) and 72.0% (CR2) compared with patients who were MRD+ (32.0% for CR1 and 44.0% for CR2) due to a higher 3y-CIR for MRD + patients. In the post-HSCT MRD measurement cohort, they found a 3y-EFS about 10.0% for patients with posMRD + by both MFC and FISH. Similar to our approach, Paras et al. [21] reported outcomes of transplanted patients combining MRD measurements before and after HSCT (days 20–40). They demonstrated that patients with the best prognosis were those with MRD- status at both time points, while patients with MRD+/posMRD- status had poorer survival. They also identified that patients with posMRD + status in the early post-transplant period had the worst survival, with comparable rates of EFS, OS and CIR to the ones reported in the present study. In their analysis, patients with posMRD + were stratified according to prior MRD status. Due to the limited sample size and the inclusion of patients with AD, which creates more subgroups for analysis, we combined all patients with posMRD + together along the general analysis, irrespective of their pre-HSCT disease status. In contrast to their study, in our sub analysis of posMRD + patients, we found that patients with transplanted with AD or MRD + who had a posMRD+, had worse survival than patients with MRD-/posMRD+. However, due to the limited sample size of posMRD + patients in our report, it is not possible to draw definitive conclusions. Additionally, we must consider that in Paras et al. report, 4 of the 18 patients with MRD-/posMRD + had > 5% blasts by MFC (8.9%, 40.9%, 71.6%, 94.1% blasts) and we have restricted the post-transplant study to patients in CR, so these may not be comparable populations. Kim et al. [24] also studied the impact of pre-HSCT and post-HSCT MRD after the first 30 days of transplant using next generation sequency (NGS) technology. They found an influence on survival for both pre- and post-HSCT MRD assessments. Persistent mutations were detected pre-HSCT in 43% of patients and in 20% on day 30 post-HSCT. When they compared patients with persistently negative MRD, persistently positive MRD, and patients with MRD+/posMRD-, they found statistically significant differences for disease-free survival (DFS) and OS. The first group have the best prognosis compared to patients with MRD+/posMRD- and specially persistently positive MRD patients. The 3y-DFS (38.1%) and 3y-OS (44.3%) of MRD+/posMRD + patients were slightly higher than those in our cohort and previous studies. This cannot be justified by the inclusion of DTA mutations, as the survival in these patients with persistent post-transplant DTA was similar, nor by choosing a 0% VAF, given the similar relapse rates reported with different cutoffs. However, we are discussing different techniques, which should not be mutually exclusive but rather complementary to better refine the prognosis of our patients [35]. Zhou et al. [23] conducted a study in transplanted patients in CR1/2 with both pre- and early post-HSCT MRD analysis (28 ± 7 days). As discussed in previous studies and in the present report, they identified the MRD-/posMRD- group as having the best prognosis. In patients with a positive pre-HSCT MRD, outcomes were adverse regardless of post-transplant MRD, although they emphasized that survival was better in patients with decreasing MRD rather than increasing MRD. Although only 7 patients had increased MRD (2 of them converted from MRD- to MRD+), all of them relapsed and died post-transplant. The post-transplant relapse rate in their group of patients with posMRD + was 80%. The two differences previously discussed regarding our study may be related to the fact that 5/16 with posMRD + had a morphological and/or cytogenetic relapse. Surprisingly, in their multivariate analysis, unlike ours and other previous studies, it was the pre-HSCT MRD, rather than post-HSCT MRD+, that showed statistically significant differences.

Unlike our report, those studies [21–24] did not include transplanted patients with AD. In our series, this group of patients had a poor prognosis despite achieving MRD negativity, with survival comparable than those with posMRD+. In the sub analysis of patients with AD/posMRD+, there was no longer survivors. Our study, consistent with previous reports [5, 6] and contrary to the findings of Araki et al. [7] regarding pre-transplant disease, indicates that a higher disease burden prior to transplantation is associated with a worse prognosis, regardless of achieving negative MRD in the early post-transplant period. This highlights the importance of disease control prior to HSCT and suggests the need for close monitoring of these patients, not only with MFC, but also potentially with other techniques such as NGS or PCR when available. The inclusion of patients with AD and not only those in CR before HSCT provides a broader perspective on the dynamics of both pre- and post-transplant measurable disease and its impact on survival. As for the post-transplant setting, we believe it makes more sense to limit the study to patients in CR (with posMRD- or posMRD+), as a higher disease burden would already indicate relapse, and it is well known that these patients have a very poor prognosis. The main limitations of this report are those inherent to the retrospective nature of our study. Moreover, the small number of posMRD + patients after transplantation has not allowed us to further subdivide this group into different subgroups based on the pre-transplant disease to draw firm conclusions in this regard.

In conclusion, pre-HSCT disease burden and early post-HSCT MRD measurements are both prognostic factors of post-HSCT survival, but the combination of both are more informative in our series. Patients with MRD + and those with AD before transplant, have an adverse outcome regardless of post-transplant MRD clearance. The prognosis of patients with posMRD + was also dismal. Patients with posMRD + in early HSCT and those with any residual disease before HSCT are patients at high risk of relapse, who could benefit from post-transplant strategies to improve outcomes.

## Electronic supplementary material

Below is the link to the electronic supplementary material.

Supplementary Material 1

## Data Availability

The data that support the findings of this study are not openly available due to reasons of confidenciality and are available from the corresponding author upon reasonable request.
