# Supplementary material for: Combination of disease burden before allogeneic transplantation and early post-transplant minimal residual disease predicts survival in patients with acute myeloid leukemia
Source: Ann Hematol. 2025 Apr 25;104(4):2469–81. doi: 10.1007/s00277-025-06325-x (PMC12053072; doi:10.1007/s00277-025-06325-x)
Supplement: Supplementary file 1 — Supplementary Material 1 [file 277_2025_6325_MOESM1_ESM.docx]

**Supplementary Table 1. Baseline characteristics in overall population, pre-HSCT disease group, post-HSCT MRD group and combined pre- and post-HSCT disease status patients.**

| **Variables** | **Total patients (n=173)** | **Pre-HSCT disease status (n=160)** | | | **Post-HSCT MRD status (n=151)** | | **Pre- and Pos-HSCT disease status (n=140)** | | | |
| --- | --- | --- | --- | --- | --- | --- | --- | --- | --- | --- |
|  |  | **MRD -(n=100)** | **MRD+**  **(n=37)** | **AD**  **(n=23)** | **posMRD- (n=136)** | **posMRD+ (n=15)** | **MRD-/posMRD- (n=82)** | **MRD+/posMRD- (n=28)** | **AD/MRD- (n=15)** | **posMRD+ (n=15)** |
| **Male sex *n* (%)** | 101 (58.4%) | 58 (58.0%)* | 27 (73.0%)* | 8 (34.8%)* | 82 (60.3%)* | 4 (26.7%)* | 49 (59.8%)* | 20 (71.5%)* | 5 (33.3%)* | 4 (26.7%)* |
| **Median age at HSCT, years (ITC range)** | 54 (45-61) | 54 (45-61) | 54 (39-63) | 56 (42-63) | 54 (42-61) | 52 (45-60) | 54 (44-60) | 54 (41-62) | 50 (39-63) | 52 (45-60) |
| **Patients ≥ 60 years at HSCT, *n* (%)** | 53 (30.6%) | 28 (28.0%) | 14 (37.8%) | 9 (39.1%) | 37 (27.2%) | 6 (40.0%) | 21 (25.6%) | 10 (35.7%) | 5 (33.3%) | 6 (40.0%) |
| **WHO 2016 classification, *n* (%)**  Recurrent genetic abnormalities  Myelodysplasia-related changes  Therapy-related  No otherwise specified  Blastic phase of MPN Ph- | 44 (25.5%)  60 (34.9%)  20 (11.7%)  42 (24.4%)  6 (3.5%) | 26 (26.3%)  28 (28.3%)  11 (11.1%)  31 (31.3%)  3 (3.0%) | 8 (21.6%)  17 (45.9%)  5 (13.5%)  6 (16.2%)  1 (2.7%) | 4 (17.4%)  13 (56.5%)  2 (8.7%)  3 (13.0%)  1 (4.3%) | 39 (28.6%)  46 (33.8%)  12 (8.8%)  36 (26.5%)  3 (2.2%) | 3 (20.0%)  5 (33.3%)  4 (26.7%)  3 (20.0%)  0 (0.0%) | 24 (29.3%)  23 (28.0%)  6 (7.3%)  27 (32.9%)  2 (2.5%) | 7 (25.0%)  12 (42.9%)  3 (10.7%)  6 (21.4%)  0 (0.0%) | 3 (20.0%)  9 (60.0%)  2 (13.3%)  1 (6.7%)  0 (0.0%) | 3 (20.0%)  5 (33.3%)  4 (26.7%)  3 (20.0%)  0 (0.0%) |
| **ELN classification, *n* (%)**  Favourable risk  Intermediate risk  Adverse risk | 31 (18.0%)  93 (54.1%)  48 (27.9%) | 20 (20.2%)  58 (58.6%)  21 (21.2%) | 5 (13.5%)  18 (48.6%)  14 (37.8%) | 1 (4.3%)  13 (56.5%)  9 (39.1%) | 27 (19.9%)  71 (52.2%)  38 (27.9%) | 3 (20.0%)  7 (46.7%)  5 (33.3%) | 17 (20.7%)  46 (56.1%)  19 (23.2%) | 5 (17.9%)  14 (50.0%)  9 (32.1%) | 1 (6.7%)  7 (46.7%)  7 (46.7%) | 3 (20.0%)  7 (46.7%)  5 (33.3%) |
| **Complex karyotype, n (%)** | 23 (14.4%) | 9 (9.1%) | 9 (24.3%) | 4 (17.4%) | 17 (12.6%) | 3 (20.0%) | 8 (9.7%) | 5 (17.9%) | 3 (20.0%) | 3 (20.0%) |
| **Monosomal karyotype, *n* (%)** | 15 (8.7%) | 5 (5.1%)* | 7 (18.9%)* | 2 (8.7%)* | 10 (7.4%) | 3 (20.0%) | 4 (4.9%) | 4 (14.3%) | 1 (6.7%) | 3 (20.0%) |
| **Patients transplanted in CR1, *n* (%)** | 118 (68.2%) | 85 (85.0%)* | 29 (78.4%)* | 0 (0.0%)* | 98 (72.1%) | 8 (53.3%) | 73 (89.0%)* | 21 (75.0%)* | 0 (0.0%)* | 8 (53.3%)* |
| **Median time from diagnosis to HSCT, months (ITC range)** | 5 (4-7) | 5 (4-7) | 4 (3-7) | 6 (4-7) | 5 (4-7) | 5 (3-9) | 5 (4-6) | 5 (3-7) | 6 (4-7) | 5 (3-9) |
| **Conditioning intensity, *n* (%)**  Myeloablative  Reduced intensity  Sequential | 91 (5.6%)*  74 (42.8%)*  8 (4.6%)* | 58 (58.0%)*  42 (42.0%)*  0 (0.0%)* | 20 (54.1%)*  16 (43.2%)*  1 (2.7%)* | 7 (30.4%)*  9 (39.2%)*  7 (30.4%)* | 77 (56.6%)  53 (39.0%)  6 (4.4%) | 5 (33.3%)  8 (53.3%)  2 (13.3%) | 51 (62.2%)*  31 (37.8%)*  0 (0.0%)* | 15 (53.6%)*  12 (42.8%)*  1 (3.6%)* | 5 (33.3%)*  5 (33.3%)*  5 (33.3%)* | 5 (33.3%)*  8 (53.3%)*  2 (13.3%)* |
| **Donor type, *n* (%)**  Related donor  Unrelated donor  Haploidentical donor | 63 (36.4%)  46 (26.6%)  64 (37%) | 38 (39.0%)  23 (23.0%)  39 (39.0%) | 17 (45.9%)  10 (27.0%)  10 (27.0%) | 5 (21.7%)  8 (34.8%)  10 (43.5%) | 54 (39.7%)  31 (22.8%)  51 (37.5%) | 6 (40.0%)  5 (33.3%)  4 (26.7%) | 34 (41.5%)  16 (19.5%)  32 (39.0%) | 14 (50.0%)  6 (21.4%)  8 (28.6%) | 3 (20.0%)  5 (33.3%)  7 (46.7%) | 6 (40.0%)  5 (33.3%)  4 (26.7%) |
| **HLA disparity, n (%)**  Full matched  Mismatch | 94 (86.2%)  15 (13.8%) | 57 (90.5%)  6 (9.5%) | 22 (81.5%)  5 (18.5%) | 9 (69.2%)  4 (30.8%) | 71 (83.5%)  14 (16.5%) | 11 (100.0%)  0 (0.0%) | 46 (88.5%)*  6 (11.5%)* | 16 (80.0%)*  4 (20.0%)* | 4 (50%)*  4 (50%)* | 11 (100.0%)*  0 (0.0%)* |
| **HCT-CI score, *n* (%)**  0  1-2  ≥3 | 89 (53.0%)  46 (27.4%)  33 (19.6%) | 51 (53.1%)  24 (25%)  21 (21.9%) | 20 (57.2%)  9 (25.7%)  6 (17.1%) | 9 (42.9%)  8 (38.1%)  4 (19.0%9 | 67 (%)*  43 (%)*  (%)* | 8 (53.3%)*  0 (0.0%)*  7 (%)* | 45 (56.9%)*  21 (26.6%)*  13 (16.5%)* | 14 (53.8%)*  9 (34.6%)*  3 (11.6%)* | 5 (38.4%)*  6 (46.2%)*  2 (15.4%)* | 8 (53.3%)*  0 (0.0%)*  7 (46.7%)* |
| **CMV serostatus donor/receptor, n (%)**  Negative/negative  Positive/Negative  Negative/Positive  Positive/Positive | 9 (5.8%)  13 (8.4%)  33 (21.3%)  100 (64.5%) | 7 (7.9%)  7 (7.9%)  19 (21.3%)  56 (62.9%) | 1 (3.2%)  3 (9.7%)  5 (16.1%)  22 (71%) | 1 (4.3%)  2 (8.7%)  5 (21.7%)  15 (65.2%) | 9 (7.3%)  11 (8.9%)  20 (16.3%)  83 (67.5%) | 0 (0.0%)  1 (7.1%)  6 (42.9%)  7 (50.0%) | 7 (9.3%)  6 (8.0%)  13 (17.3%)  49 (65.4%) | 5 (21.7%)  2 (8.7%)  3 (13.0%)  17 (73.9%) | 1 (4.2%)  2 (8.3%)  2 (8.3%)  19 (79.2%) | 0 (0.0%)  1 (7.1%)  6 (42.9%)  7 (50.0%) |
| **CD34+^6^/kg, median (range)** | 5.5 (4.5-6.3) | 6 (4.5-6.4)* | 5 (4-6)* | 5.4 (4.4-6.2)* | 5.6 (4.6-6.6) | 4.6 (3.6-6.0) | 6.0 (4.7-6.5) | 5.0 (4.2-6) | 5.0 (4.2-6.0) | 4.6 (3.6-6.0) |

*p value <0.05* *p* value < 0.05; CR, complete remission; CMV, cytomegalovirus; ELN, European LeukemiaNet; GVHD, graft-vs-host disease; HCT-CI, haematopoietic cell transplantation-specific comorbidity index; HSCT, haematopoietic stem cell transplantation; MPN Ph-, myeloproliferative neoplasm Philadelphia negative; MRD, minimal residual disease; NOS, not otherwise specified; WHO, World Health Organisation.

**Supplementary Figure 1. Event-free survival (S1A) and overall survival (S1B) on patients with known preHSCT disease who were posMRD+.**

Estimates of (left) EFS and (right) OS after HSCT for patients with AML with posHSCT MRD+ according to preHSCT, shown individually for MRD-/posMRD+ (n=5), MRD+/posMRD4 (n=4), AD/posMRD+ (n=4) respectively. AD, active disease; EFS, event-free survival; HSCT, haematopoietic stem cell transplantation; MRD, minimal residual disease; OS, overall survival

**Supplementary figure 2. Non-relapse mortality according to preHSCT status (SF2A), posHSCT MRD (SF2B) and combined pre and posHSCT disease burden (SF2C)**

Estimates of NRM after HSCT for patients with AML according to preHSCT (up-left), shown individually for MRD- (n=100), MRD+ (n=37) and AD (n=23) respectively. Estimates of NRM after HSCT for patients with AML according to posHSCT (up-right) and combined pre and posHSCT disease burden (down-left), shown individually for posMRD- (n=136) and posMRD+ (n=15) respectively. Estimates of NRM after HSCT for patients with AML according to pre and posHSCT disease burden (down-left),according to combined preHSCT and posHSCT, shown individually for MRD-/posMRD- (n=82), MRD+/posMRD- (n=28), AD/posMRD- (n=15) and posMRD+ (n=15) respectively. There were no differences in NRM according to preHSCT disease status, posHSCT or combined pre and posHSCT disease status (preHSCT disease status: 3y-NRM 24.5% in MRD- vs 20.0% in MRD+ vs 22.5% in AD patients; posHSCT MRD status: 3y-NRM 18.0% in posMRD- vs 13.0% in posMRD+; combined pre and posHSCT disease status: 3y-NRM: 17.5% in MRD-/MRD- vs 15.0% in MRD+/MRD- vs 18.0% in AD/MRD- and 13.0% in posMRD+ respectively). AD, active disease; HSCT, haematopoietic stem cell transplantation; MRD, minimal residual disease; NRM, non-relapse mortality.

**Supplementary figure 1**

**
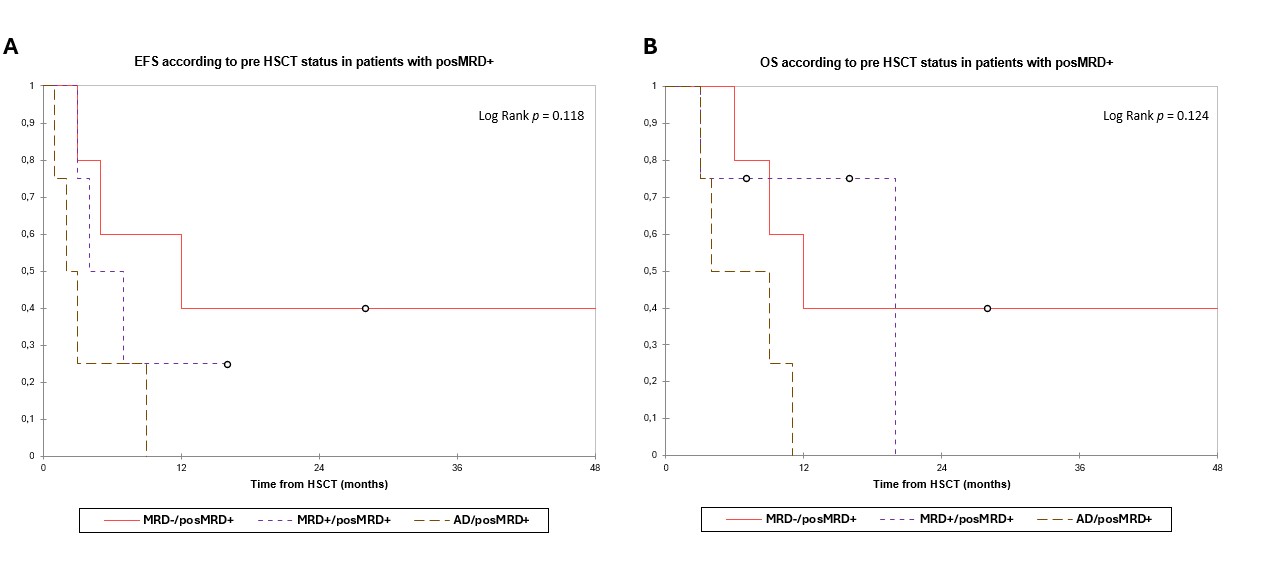
**

**
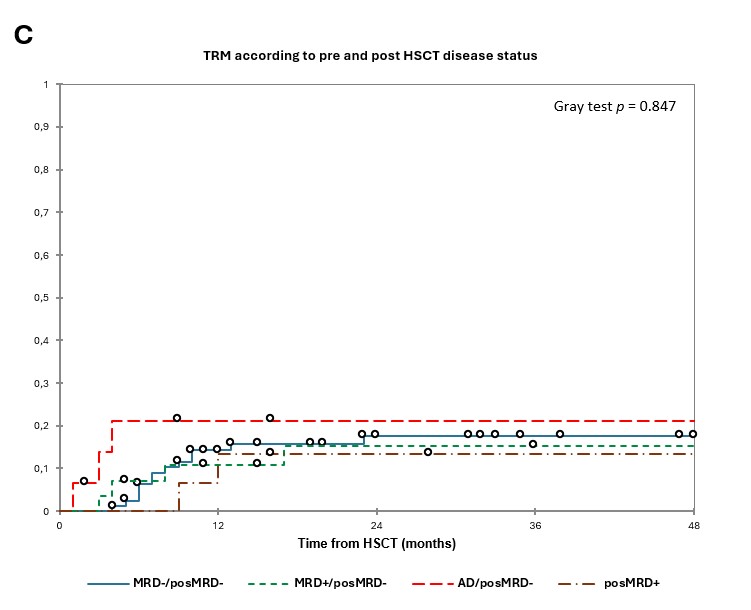

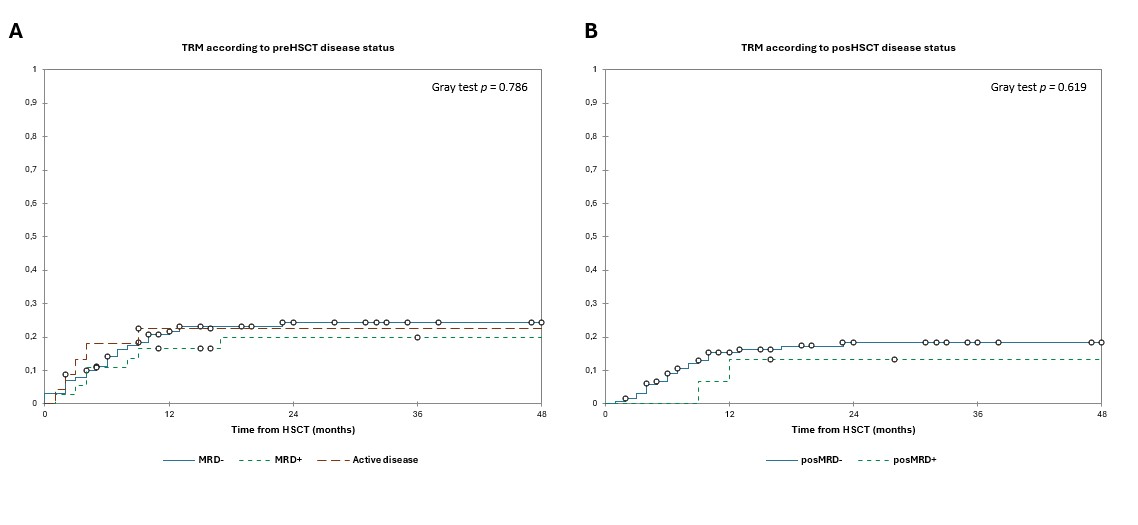
Supplementary figure 2**
